# Quorum Sensing Activity of *Hafnia alvei* Isolated from Packed Food

**DOI:** 10.3390/s140406788

**Published:** 2014-04-14

**Authors:** Jia-Yi Tan, Wai-Fong Yin, Kok-Gan Chan

**Affiliations:** Division of Genetics and Molecular Biology, Institute of Biological Sciences, Faculty of Science, University of Malaya, Kuala Lumpur 50603, Malaysia; E-Mails: chloe_tjy@yahoo.com (J.-Y.T.); yinwaifong@yahoo.com (W.-F.Y.)

**Keywords:** *Hafnia alvei*, mass spectrometry, *N*-acylhomoserine lactone, *N*-(3-oxohexanoyl) homoserine lactone, *N*-(3-oxooctanoyl) homoserine lactone, quorum sensing, fish products, food spoilage, food microbiology, food safety

## Abstract

Quorum sensing (QS) is a mechanism adopted by bacteria to regulate expression of genes according to population density. *N*-acylhomoserine lactones (AHLs) are a type of QS signalling molecules commonly found in Gram-negative bacteria which have been reported to play a role in microbial spoilage of foods and pathogenesis. In this study, we isolated an AHL-producing *Hafnia alvei* strain (FB1) from spherical fish pastes. Analysis via high resolution triple quadrupole liquid chromatography/mass spectrometry (LC/MS) on extracts from the spent supernatant of *H. alvei* FB1 revealed the existence of two short chain AHLs: *N*-(3-oxohexanoyl) homoserine lactone (3-oxo-C6-HSL) and *N*-(3-oxo- octanoyl) homoserine lactone (3-oxo-C8-HSL). To our knowledge, this is the first report of the production of AHLs, especially 3-oxo-C8-HSL, by *H. alvei*.

## 1. Introduction

Quorum sensing (QS) is a means of cell-to-cell communication adopted by a number of bacterial species that is based on the release and detection of signalling molecules called autoinducers (AIs) [1]. *N*-acylhomoserine lactones (AHL) are one type of these molecules commonly produced by Gram-negative bacteria. A constant diffusion of AHLs across the cell membrane along a concentration gradient enables certain bacterial genes or gene clusters to be regulated according to population density [2].

The focus in QS studies had largely been placed on its role in bacterial pathogenesis [3]. In recent years, the association of QS with microbial food spoilage, an event causing severe economic losses in the food industry as well as public health problems [4], has gained researchers' interest. In addition, biofilm formation, a potential source of chronic contamination attached to food processing surfaces, is also known to be under regulation of QS [5]. It has been reported that there is a correlation between the levels of AHLs detectable in spoilt foods, the growth of spoilage bacteria, as well as the expression of some proteolytic phenotypes [6].

*Hafnia alvei*, a Gram-negative, motile, flagellated, facultative anaerobic bacillus, is known to be among the AHL-producing *Enterobacteriaceae* species most commonly isolated from vacuum-packed chilled meat samples [7], and an opportunistic pathogen [8]. Results from most studies agree that *H. alvei* produces *N*-(3-oxohexanoyl) homoserine lactone (3-oxo-C6-HSL), and the QS activity of this species has been associated with both food spoilage and biofilm formation [9]. In this study we present the mass spectrometry profiling of AHLs produced by *H. alvei* strain FB1 isolated from vacuum-packed, refrigerated spherical fish paste (in the form of meatballs), a common Malaysian food popular in Southern China and South East Asia. The production of fish paste meatballs involves mashing and mixing of the ingredients, which brings them into frequent contact with food processing surfaces. This food sample was chosen in order to study the food-associated, AHL-producing bacterial cells from the contaminated surface of the food matrices, a unique microenvironment in which the signalling molecules could easily accumulate with high density, making the resulting QS-modulated traits such as spoilage and biofilm formation more prominent.

## 2. Experimental Section

### 2.1. Sample Collection and Processing

Vacuum-packed, refrigerated spherical fish paste samples of different brands were collected from a local supermarket in Malaysia. The samples were processed immediately upon reaching the laboratory. Five grams of the stomached samples were incubated in Brain Heart Infusion (BHI) broth (50 mL) overnight at 37 °C with shaking (200 rpm).

### 2.2. Isolation and Identification of Bacterial Strains

A tenfold serial dilution of 10^−1^, 10^−2^, 10^−3^, 10^−4^, and 10^−5^ was made from the overnight cultures, each dilution was spread on MacConkey (MAC) agar plates. Bacteria isolated were then identified via a Bruker MALDI Biotyper System (Bruker, Daltonik GmbH, Leipzig, Germany) [10] using the extraction method as provided by the manufacturer. The results were validated with 16S rDNA PCR using primer sequences and PCR conditions previously described by Chan *et al.* [11]. Phylogenetic analysis was carried out using MEGA 5.2 software [12] by comparing the 16S rDNA sequence of *H. alvei* FB1 to the closely related sequences available in the GenBank database (http://www.ncbi.nlm.nih.gov/genbank/).

### 2.3. AHL Detection of Bacteria Isolates

A rapid screening for short chain AHL production was performed on all bacterial isolates by cross streaking with biosensor *Chromobacterium violaceum* CV026. *Erwinia carotovora* GS101 and *E. carotovora* PNP22 were used as positive and negative controls, respectively [13].

### 2.4. AHL Extraction

AHL were extracted thrice from 100 mL of overnight LB broth culture (buffered with 50 mM of 3-[*N*-morpholino]propanesulfonic acid, MOPS, pH 5.5) [14] of *H. alvei* FB1 with acidified ethyl acetate (0.1% (v/v) glacial acetic acid). The extracts were dried in sterile microcentrifuge tubes and stored for at −20 °C.

### 2.5. AHL Identification via Triple Quadrupole LC/MS

AHL extracts were reconstituted in 1 mL of acetonitrile and 100 μL of the reconstituted extracts was loaded for LC/MS analysis. Parameters applied and instrument settings were as described by Lau *et al.* [15]. Ten synthetic AHLs and oxo-derivatives of known carbon chain lengths were used as the standards for comparison. Thin layer chromatography was performed as a confirmation test alongside the LC/MS analysis, according to the method described by Chen *et al.* [16], using synthetic 3-oxo-C6-HSL (0.1 μg/μL) and N-(3-oxooctanoyl) homoserine lactone (3-oxo-C8-HSL, 5 μg/μL) as standards.

## 3. Results and Discussion

**Figure 1.**
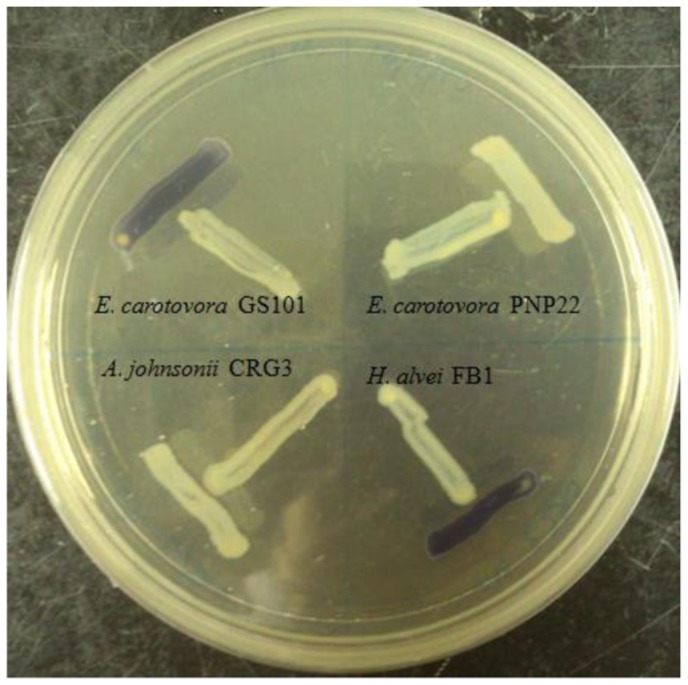
Screening for AHL production using *C. violaceum* CV026 cross streaking with *E. carotovora* GS101 and PNP22 as positive and negative controls, respectively. *H. alvei* FB1 was found to induce the violacein production in CV026. The biosensor did not respond to the non-AHL-producing isolate, *Acinetobacter johnsonii* CRG3.

Four *Enterobacteriaceae* strains were isolated from a same spherical fish paste sample and identified, only *H. alvei* FB1 showed positive results after a 24 h incubation in the preliminary screening with CV026 (Figure 1).

**Figure 2.**
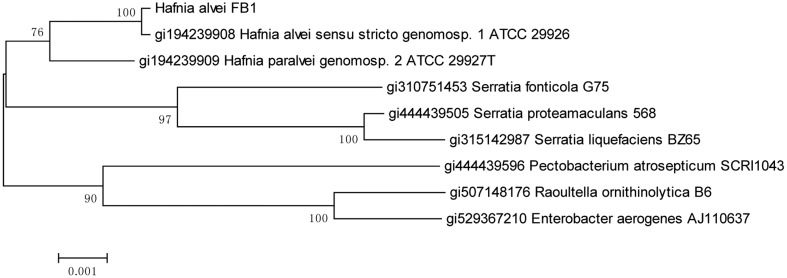
The 16S rDNA phylogenetic analysis of isolate FB1. The optimal tree with the sum of branch length = 0.03685960 is shown. The percentages of bootstrap test (1,000 replicates) are shown next to the branches. The tree is drawn to scale, with branch lengths in the same units as those of the evolutionary distances used to infer the phylogenetic tree. The analysis involved 9 nucleotide sequences. Codon positions included were 1st + 2nd + 3rd + Non-coding. All positions containing gaps and missing data were eliminated. There were a total of 1,485 positions in the final dataset.

Bacterial identification using MALDI-TOF MS platform has identified the AHL-producing isolate as the species *H. alvei* with a high confidence score of 2.655 (the highest score value being 3.000). This identification was consistent with the result of phylogenetic analysis of the 16S rDNA on MEGA (Figure 2) where the evolutionary history was inferred using the Neighbour-Joining method [17]. The percentage of replicate trees in which the associated taxa clustered together in the bootstrap test (1,000 replicates) are shown next to the branches [18]. The evolutionary distances were computed using the Maximum Composite Likelihood [19] method and are in the units of the number of base substitutions per site.

The genus *Hafnia* originally contained only one recognized species, namely *H. alvei*, which in fact consisted of the new species category *H. paralvei* and some of the strains previously designated as the now obsolete *Obesumbacterium proteus*. The name *H. alvei sensu lato* was even included for an enteropathogen later identified as a new species, *Escherichia albertii* [20]. Our phylogenetic analysis results showed that FB1 belongs to *H. alvei*.

**Figure 3.**
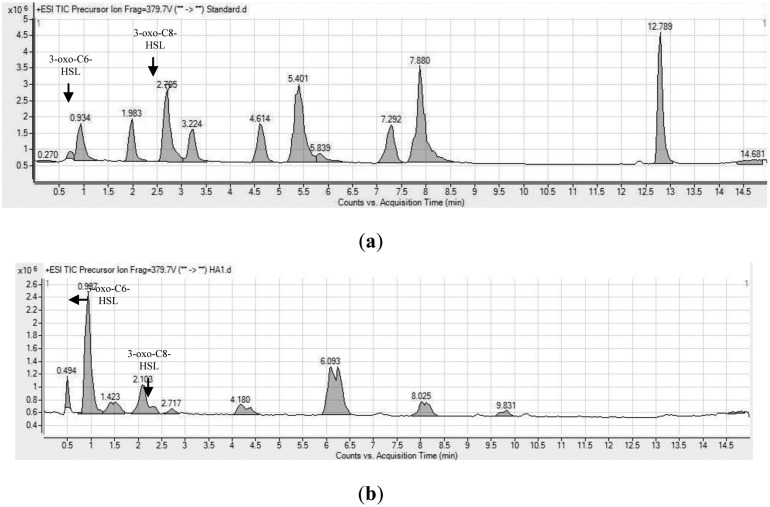
Chromatograms showing the peaks of targeted AHLs (3-oxo-C6-HSl, 3-oxo-C8-HSL, marked by arrows) (**a**) Synthetic AHL standards (1 ppm each); (**b**) AHLs extracted from the cell-free supernatant of *H. alvei* FB1

**Figure 4.**
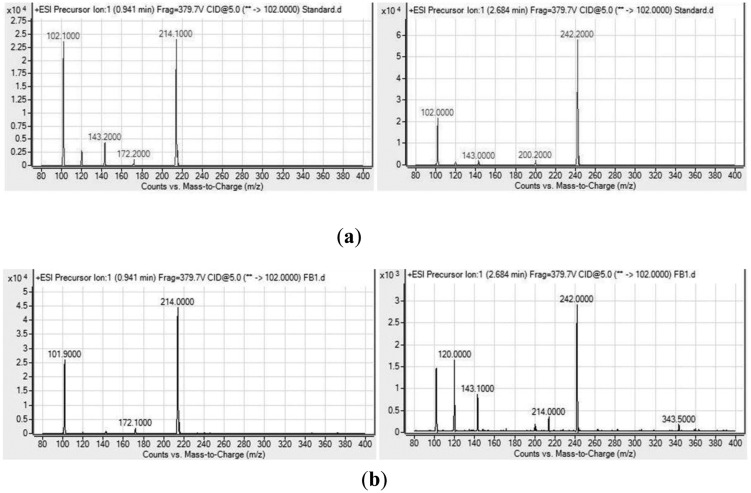
Mass spectra of (**a**) Synthetic AHLs standard; (**b**) AHLs extracted from the cell-free supernatant of *H. alvei* FB1, showing the peaks of targeted AHLs: 3-oxo-C6-HSL (*m/z* 214) and 3-oxo-C8-HSL (*m/z* 242) along with the product ion peaks (*m/z* 102.0).

**Figure 5.**
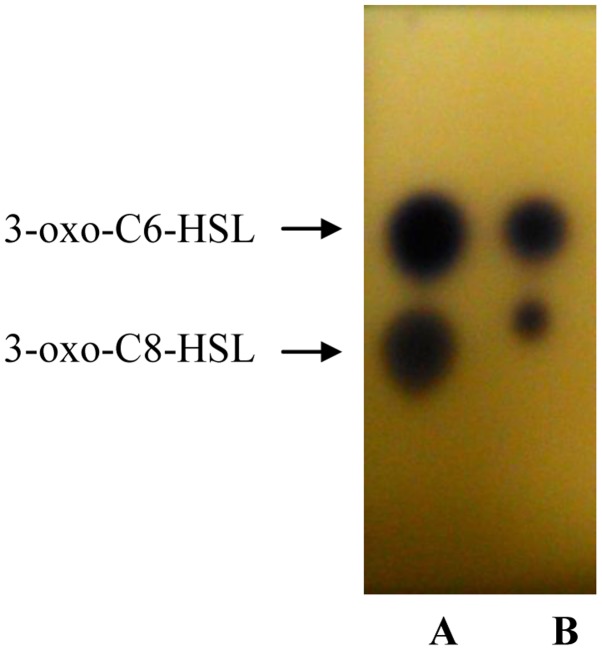
TLC separation of AHLs present in extract of the spent culture supernatant of *H. alvei* FB1, visualised with agar seeded with *C. violaceum* CV026 cells. Lana A: Synthetic AHL standards (marked by arrows); Lane B: AHLs extracted from *H. alvei* FB1

Analysis of the spectra generated on the LC/MS platform by comparing to a series of ten synthetic AHLs and oxo-derivatives with known chain lengths revealed that *H. alvei* FB1 produced two types of AHLs: 3-oxo-C6-HSL (*m/z* 214) and 3-oxo-C8-HSL (*m/z* 242). The charge-to-mass ratio (*m/z*) values of these detected compounds are consistent with those reported by Ortori *et al.* [21]. The total ion chromatograms and mass spectra are shown in Figures 3 and 4, respectively. This finding was supported by the TLC results (Figure 5), which showed the formation of two well-separated purple spots with retention factors (R*_f_*) similar to the corresponding synthetic AHLs (R*_f_* 10.0 for 3-oxo-C6-HSL and R*_f_* 14.2 for 3-oxo-C8-HSL). In the mass spectral analysis, the amount of 3-oxo-C6-HSL (ion abundance 44594.28, 100% of the base peak abundance) produced was much higher than that of 3-oxo-C8-HSL (ion abundance 2922.20, 100% of the base peak abundance) (Figure 3).

Being a rather heterogeneous cluster, *H. alvei* has been isolated from a wide range of sources apart from foods, including soil, water, and a variety of animals [8]. According to the previous publications [7,9,22], 3-oxo-C6-HSL seems to be the form of signalling molecule commonly detected throughout the species [5]; production of 3-oxo-C8-HSL by *H. alvei*, however, has not been documented before, and to the best of our knowledge, this is the first paper reporting the detection of 3-oxo-C8-HSL produced by *H. alvei* confirmed by a triple quadrupole LC/MS platform.

From the previous reports we know that 3-oxo-C6-HSL was the most common signalling molecule detected in foods [23], and a report by Christensen *et al.* has demonstrated its role in regulating the expression of LipB, which was required for the lipolytic and proteolytic activities in *Serratia proteamaculans* [7]. Being a close relative to the genus *Serratia*, it is not unlikely that the same functional role applies in *H. alvei* , but further work in needed to confirm this speculation.

On the other hand, 3-oxo-C8-HSL is not among the forms of AHL usually detected in foods. It is, however, often detected and most intensively studied in plant-associated bacteria [24–26]. Presence of this molecule has been linked to the virulence genes in *Pseudomonas aeruginosa*, an opportunistic human pathogen [27], as well as *Yersinia ruckeri*, a fish pathogen [28]. Whether 3-oxo-C8-HSL has a role in pathogenesis of *H. alvei* FB1, and it contributes to the survival or colonization of the strain in its environment of origin, remain questions to be solved in more in-depth studies.

## 4. Conclusions

In this study we reported for the first time the production of 3-oxo-C8-HSL by *H. alvei*. The finding signifies the diversity of QS system within a potential food spoilage and opportunistic pathogen, which is indicative to the existence of differential regulation mechanisms for optimal survival in different environments. Our future study will be focused on the downstream regulations of QS system in *H. alvei*, the types and quantities of AHLs produced in response to changes in the environment, and their significance in food industry and control of food spoilage using anti-QS strategy [29–33].

## References

[1] Miller M.B., Bassler B.L. (2001). Quorum sensing in bacteria. Annu. Rev. Microbiol..

[2] Parsek M.R., Greenberg E.P. (2000). Acyl-homoserine lactone quorum sensing in gram-negative bacteria: A signaling mechanism involved in associations with higher organisms. Proc. Natl. Acad. Sci. USA.

[3] De Kievit T.R., Iglewski B.H. (2000). Bacterial quorum sensing in pathogenic relationships. Infect. Immun..

[4] Kumar C.G., Anand S.K. (1998). Significance of microbial biofilms in the food industry: A review. Intl. J. Food Microbiol..

[5] Bai A.J., Rai V.R. (2011). Bacterial quorum sensing and food industry. Compr. Rev. Food Sci. Food Saf..

[6] Jay J.M., Vilai J.P., Hughes M.E. (2002). Profile and activity of the bacterial biota of ground beef held from freshness to spoilage at 5–7 °C. Food Microbiol..

[7] Bruhn J.B., Christensen A.B., Flodgaard L.R., Nielsen K.F., Larsen T.O., Givskov M., Gram L. (2004). Presence of acylated homoserine lactones (AHLs) and AHL-producing bacteria in meat and potential role of ahl in spoilage of meat. Appl. Environ. Microbiol..

[8] Janda J.M., Abbott S.L. (2006). The genus *Hafnia*: From soup to nuts. Clin. Microbiol. Rev..

[9] Viana E.S., Campos M.E., Ponce A.R., Mantovani H.C., Vanetti M.C. (2009). Biofilm formation and acyl homoserine lactone production in *Hafnia alvei* isolated from raw milk. Biol. Res..

[10] Seng P., Drancourt M., Gouriet F., La Scola B., Fournier P.E., Rolain J.M., Raoult D. (2009). Ongoing revolution in bacteriology: Routine identification of bacteria by matrix-assisted laser desorption ionization time-of-flight mass spectrometry. Clin. Infect. Dis..

[11] Chan K.G., Tiew S.Z., Ng C.C. (2007). Rapid isolation method of soil bacilli and screening of their quorum quenching activity. Asia Pac. J. Mol. Biol. Biotech..

[12] Tamura K., Peterson D., Peterson N., Stecher G., Nei M., Kumar S. (2011). Mega5: Molecular evolutionary genetics analysis using maximum likelihood, evolutionary distance, and maximum parsimony methods. Mol. Biol. Evol..

[13] McClean K.H., Winson M.K., Fish L., Taylor A., Chhabra S.R., Camara M., Daykin M., Lamb J.H., Swift S., Bycroft B.W. (1997). Quorum sensing and *Chromobacterium violaceum*: Exploitation of violacein production and inhibition for the detection of *N*-acylhomoserine lactones. Microbiology.

[14] Yates E.A., Philipp B., Buckley C., Atkinson S., Chhabra S.R., Sockett R.E., Goldner M., Dessaux Y., Cámara M., Smith H. (2002). *N*-acylhomoserine lactones undergo lactonolysis in a ph-, temperature-, and acyl chain length-dependent manner during growth of *Yersinia pseudotuberculosis* and *Pseudomonas aeruginosa*. Infect. Immun..

[15] Lau Y.Y., Sulaiman J., Chen J.W., Yin W.F., Chan K.G. (2013). Quorum sensing activity of *Enterobacter asburiae* isolated from lettuce leaves. Sensors.

[16] Chen J.W., Koh C.L., Sam C.K., Yin W.F., Chan K.G. (2013). Short chain *N*-acyl homoserine lactone production by soil isolate *Burkholderia* sp. strain A9. Sensors.

[17] Saitou N., Nei M. (1987). The neighbor-joining method: A new method for reconstructing phylogenetic trees. Mol. Biol. Evol..

[18] Felsenstein J. (1985). Confidence limits on phylogenies: An approach using the bootstrap. Evolution.

[19] Tamura K., Nei M., Kumar S. (2004). Prospects for inferring very large phylogenies by using the neighbor-joining method. Proc. Natl. Acad. Sci. USA.

[20] Huys G., Cnockaert M., Janda J.M., Swings J. (2003). *Escherichia albertii* sp. Nov., a diarrhoeagenic species isolated from stool specimens of Bangladeshi children. Int. J. Syst. Evol. Microbiol..

[21] Ortori C.A., Dubern J.F., Chhabra S.R., Cámara M., Hardie K., Williams P., Barrett D.A. (2011). Simultaneous quantitative profiling of *N*-acyl-L-homoserine lactone and 2-alkyl-4(1h)-quinolone families of quorum-sensing signaling molecules using LC-MS/MS. Anal. Bioanal. Chem..

[22] Pinto U.M., de Souza Viana E., Martins M.L., Vanetti M.C.D. (2007). Detection of acylated homoserine lactones in gram-negative proteolytic psychrotrophic bacteria isolated from cooled raw milk. Food Control.

[23] Skandamis P.N., Nychas G.J. (2012). Quorum sensing in the context of food microbiology. Appl. Environ. Microbiol..

[24] Zhu J., Beaber J.W., Moré M.I., Fuqua C., Eberhard A., Winans S.C. (1998). Analogs of the autoinducer 3-oxo-octanoyl-homoserine lactone strongly inhibit activity of the trar protein of agrobacterium tumefaciens. J. Bacteriol..

[25] Cha C., Gao P., Chen Y.C., Shaw P.D., Farrand S.K. (1998). Production of acyl-homoserine lactone quorum-sensing signals by gram-negative plant-associated bacteria. Mol. Plant Microbe Interact..

[26] Elasri M., Delorme S., Lemanceau P., Stewart G., Laue B., Glickmann E., Oger P.M., Dessaux Y. (2001). Acyl-homoserine lactone production is more common among plant-associated *Pseudomonas* spp. than among soilborne *Pseudomonas* spp. Appl. Environ. Microbiol..

[27] Pearson J.P., Gray K.M., Passador L., Tucker K.D., Eberhard A., Iglewski B.H., Greenberg E.P. (1994). Structure of the autoinducer required for expression of *Pseudomonas aeruginosa* virulence genes. Proc. Natl. Acad. Sci. USA.

[28] Kastbjerg V.G., Nielsen K.F., Dalsgaard I., Rasch M., Bruhn J.B., Givskov M., Gram L. (2007). Profiling acylated homoserine lactones in *Yersinia ruckeri* and influence of exogenous acyl homoserine lactones and known quorum-sensing inhibitors on protease production. J. Appl. Microbiol..

[29] Koh C.-L., Sam C.-K., Yin W.-F., Tan L.Y., Krishnan T., Chong Y.M., Chan K.-G. (2013). Plant-derived natural products as sources of anti-quorum sensing compounds. Sensors.

[30] Hong K.-W., Koh C.-L., Sam C.-K., Yin W.-F., Chan K.-G. (2012). Quorum quenching revisited—from signal decays to signalling confusion. Sensors.

[31] Yin W.-F., Tung H.-J., Sam C.-K., Koh C.-L., Chan K.-G. (2012). Quorum quenching *Bacillus sonorensis* isolated from soya sauce fermentation brine. Sensors.

[32] Wong C.-S., Koh C.-L., Sam C.-K., Chen J.W., Chong Y.M., Yin W.-F., Chan K.-G. (2013). Degradation of bacterial quorum sensing signaling molecules by the microscopic yeast *Trichosporon loubieri* isolated from tropical wetland waters. Sensors.

[33] Chong T.-M., Koh C.-L., Sam C.-K., Choo Y.-M., Yin W.-F., Chan K.-G. (2012). Characterization of quorum sensing and quorum quenching soil bacteria isolated from Malaysian tropical montane forest. Sensors.

